# Impact on Tuberculosis Notification During COVID-19 Pandemic in India: A Narrative Review

**DOI:** 10.7759/cureus.44087

**Published:** 2023-08-25

**Authors:** Sweta Sahu, Nikhilesh Nagtode

**Affiliations:** 1 Epidemiology and Public Health, Datta Meghe Institute of Higher Education & Research, Wardha, IND; 2 Community Medicine, Datta Meghe Institute of Higher Education & Research, Wardha, IND

**Keywords:** ctd(central tb division), nikshay portal, health services in india, tb notification, covid-19

## Abstract

Various programs are being weakened due to the COVID-19 pandemic, and the tuberculosis (TB) program is no exception. TB case detection and notification is one of the worst affected areas. The study aims to assess India's TB reporting status during this pandemic and find possible solutions. Data analysis has been obtained from the India TB notification open-source database. Relevant literature research has been done to determine the measures based on various efforts made by different Indian states. There was a review of all TB notifications in 2019, 2020, and 2021 and a deficiency in notifications. Between 2019 and 2021, the country's TB notification ratio experienced a significant adaptation. In 2020, all states reported a decline in private and public TB case reports. In the nation, only a few private TB notifications were lost. In April 2020, there were the fewest notifications, which began to decline in February 2020. When states began implementing cutting-edge programs like the Integrated TB COVID Case Search and Active Case Finding (ACF), the notification trend improved in May 2020. The notifications of TB cases decreased significantly due to the present COVID-19 pandemic, which has consequences for the disease's stealthy spread throughout homes and communities. However, the situation may be better with an integrated strategy for managing TB-COVID cases.

## Introduction and background

Across the globe, the COVID-19 pandemic has led to more than 6 million deaths. Research and development have occurred in tuberculosis (TB) treatment, prevention, and new technologies. This worldwide strategy has already influenced the 2022 G20 TB discussions and the worldwide Fund Investment Case [1,2]. The COVID-19 epidemic has exposed and drawn attention to weaknesses in India's public health system. The COVID-19 pandemic response has shown systemic failures in our preparedness and response [3,4]. COVID-19 impacted many effective health programs, including the TB program [5,6]. TB detection and eradication benefits have decreased due to the COVID-19 pandemic's compromise of TB diagnosis, treatment, and prevention [7]. Three million COVID-19 cases had been reported globally as of the End of April 2020. With a population of 300 million people, the United States has one million cases of COVID-19 [8]. The TB pathogen Mycobacterium tuberculosis (M. tb) is responsible for a large ratio of deaths globally [9].

Recent research evidence suggests that reduced cell-mediated susceptibility caused by COVID-19, a coronavirus disease, increases the activation of latent TB, posing a significant challenge to the goal of eliminating TB by 2035 Goes [10]. A global plan to end TB has been drawn up, with a plan to end TB as a public health challenge by 2023-2030. The government provides a specific assessment of the financial resources needed in the fight against TB, as well as an action plan for key priorities. The earlier version of the Global Plan, which was based on the global commitments of Member States at the 2018 United Nations High-Level Meeting (UNHLM) on TB, detailed the resources needed to prioritize action for 2018-2022, and this new version expands on them. To incorporate this, resources would need to be removed from this overall design [11,12]. 

In India, the TB program was changed to the National TB Elimination Programme (NTEP) in 2019 to eradicate the disease five years before the Sustainable Development Goals (SDGs) deadline 2025. One of the biggest reasons for death and morbidity on the globe is tuberculosis. One of the key elements contributing to the spread of the TB disease has been identified as household contact with active tuberculosis (TB) patients [13]. While both TB and COVID-19 are contagious through intimate human contact, the specific mode of transmission differs, partly because the two diseases are treated differently regarding infection control strategies. After a TB patient sneezes, coughs, sings, or screams, TB bacilli droplet nuclei continue to be suspended in the air for several hours, and people can become ill if inhaled [14,15]. While the expected outcome and consequences can occasionally vary, the condition is often characterized by early signs and symptoms comparable to those of related viral diseases such as Middle East respiratory syndrome (MERS), severe acute respiratory syndrome (SARS), and influenza. There is a severe lack of experience with concurrent TB and COVID-19 [16]. Globally, a three-month lockout and 10-month recovery period might lead to an extra 6.3 million TB infections and 1.4 million TB fatalities between 2020 and 2025 [9,17]. It is estimated that one-third of the world's population has latent TB, which can become active in immune-compromised individuals with co-morbidities. COVID-19 has challenged the infrastructural components of the health system, such as diagnostic equipment and the staff, which have been redirected towards COVID-19 and diverted by competing objectives, such as TB [18]. Eliminating TB in India would be crucial as the country changes from the Millennium Development Goals (MDGs) to the more determined and universal Sustainable Development Goals (SDGs), boosting our determination with a comprehensive approach to health. The period between 2020 and 25 has become extremely important as the Honorable PM issued a call to action to end TB by 2025, five years earlier than the SDGs aim [19]. During this time, considerable progress must be made toward ending TB.

The program in India has been rebranded as the National TB Elimination Program (NTEP) and is carrying out TB prevention and control operations in a mission mode to End TB by 2025. The 4 strategic pillars of "Detect - Treat - Prevent - Build" (DTPB) are included to eliminate tuberculosis [2,20]. There has been a 26% fall in reported TB cases associated with the same period last year, highlighting the global impact of the COVID-19 pandemic [19].

## Review

Method 

Search Methodology 

**Figure 1:**
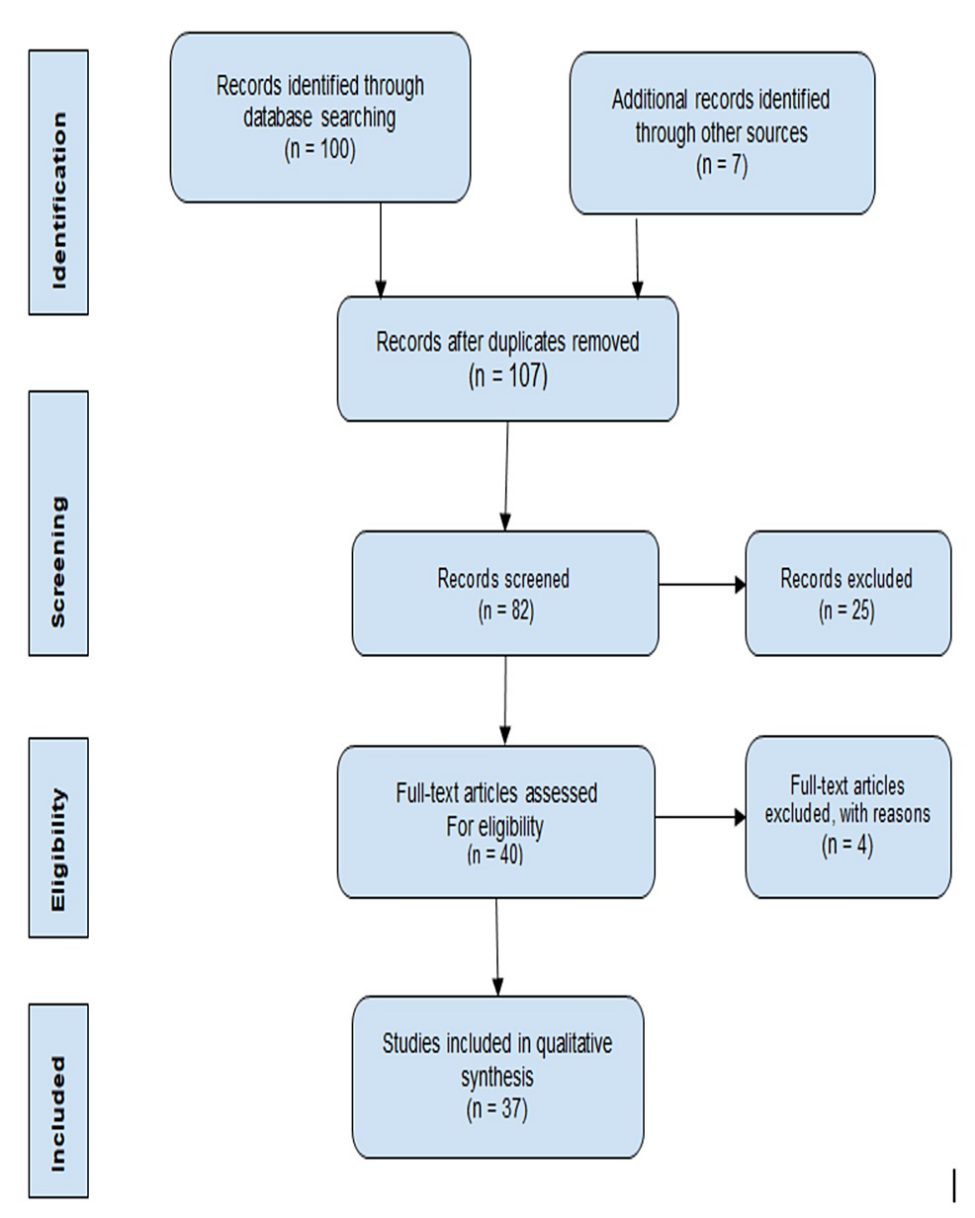
PRISMA flow diagram for literature search

This paper discusses the impact of TB notification during the COVID-19 pandemic. Our report is based on previously presented domestic and worldwide literature on a search of multiple sources. In which duration have searched for 3-5 years. We explored several websites for recent related statistics and literature in Google Scholar, Sci-Hub, PubMed, and Research Gate. Also, we used the government website like the E-notification system (Nikshay portal). Using the keywords COVID-19, TB notification, pandemic duration, health services in India, Nikshay portal, and Central TB Division (CTD), we found over 100 articles, some of which were from government websites. Finally, we included 33 articles and 4 government websites for citations in the article. See Figure 1 below.

Nikshay is the CTD of the Indian Government's Ministry of Health and Family Welfare's online portal, which allows the public to report and monitor TB cases. This system makes access to TB case Notifications in real time in India's public and private sectors possible. From March 2020 to April 2021, TB case notification data was collected from the Nikshay by day and place (national/territory). More than half of the overall difference in predicted cases recorded, as well as the state of Chhattisgarh [21], is due to the epidemic's presence in four states (Rajasthan, Maharashtra, Uttar Pradesh, and Madhya Pradesh). Comparisons between January and December were done year over year. Each region's total TB notification (public-private) was evaluated independently. The percentage reduction in TB notification reports between January 1 and July 31, 2020, and January 1 and July 31, 2019, was estimated by comparing the notification data in each state [22].

There was a decline in TB reports in India in 2019 or 2020 (the shutdown era). Was COVID the reason for less notification? The Covid pandemic has further worsened the performance of many programs like the TB program. The 1st case was notified of COVID-19 in India on January 27, 2020 [23], and reports began to decline in February 2020. The shortest information happened in April, and on March 24, a national lockdown was announced in India. These temporalities suggest that the decline in TB notification was due to the direct and indirect effects of the COVID-19 pandemic [24].

One of the precautions used at the beginning of the epidemic was to isolate for at least 14 days or more, but if symptoms persist, TB is present. The symptoms and signs of pulmonary TB are associated with respiratory viral infections, such as COVID-19, which intervals the diagnosis of TB. Additionally, being close to one another increases the chance of TB transmission among household members [25].

Action plan for TB and COVID integration: It is impossible and not recommended during this epidemic to switch attention and resources from COVID-19 to TB. However, it is possible to find points of overlap and combine efforts to achieve mutual benefit. Establishing a system for hospital and community-based cough screening is only one example of this kind of synergy. A testing technique was created to rule out TB following or in conjunction with testing for COVID [26]. For screening, isolation, quick tracking, and testing of individuals exhibiting coughing symptoms, airborne infection-cum-TB screening was created in healthcare institutions [22].

Discussion

Effect of Public and Private Notification

**Figure 2:**
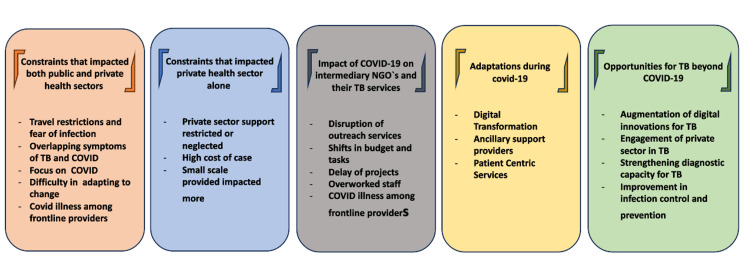
During COVID-19, there will be chances for collaboration and rethinking TB treatment in the private health sector. Authors own work.

The WHO advised associate states to adopt government and whole-of-society strategies in comeback to the COVID-19 pandemic. It examined the potential impact of TB and mitigation measures [27,28]. Although (PPP)public-private partnerships could support overburdened health systems, the nation was unclear on the best ways to engage the business sector in its efforts to combat COVID-19 at a national level [29,30]. However, it has traditionally been viewed as a stand-alone organization that operates in silos. The business sector came forward to help public efforts during the pandemic. Opened its doors to help manage COVID-19 public health professionals [11,27]. This also holds applicable for TB. This fast evaluation was co-written by fifteen intermediate agency representatives from the Big Seven nations of Pakistan, Nigeria, Myanmar, Bangladesh, India, the Philippines, and Indonesia to provide information in this area [24,29]. In addition to the effect of the COVID-19 pandemic on TB PPM among private-sector TB care providers, TB patients, and agencies, many post-pandemic adjustments were adversely affected, and plans to increase TB care were postponed. The findings are summarized in Fig 2.

On March 20, 2020, the WHO issued a new statement: "COVID-19: Attentions for tuberculosis (TB) care services- As the world works together to fight the COVID-19 pandemic, it is imperative to confirm that critical services and processes for managing chronic health concerns remain to preserve the exists of people with TB and other diseases or health conditions. Health services, especially national TB programs, must be actively involved in providing an effective and rapid response to COVID-19 and maintaining TB services [31,32].

Nikshay e-Platform

TB PPM (Public-private mix)among private-sector TB care providers and agencies was found to be affected by the COVID-19 pandemic as well as a lack of care in multiple disease plans [6,18,21,26]. TB notifications every year between March 20 and July 3 increased, but in 2020, TB due to COVID-19, less than half of the reported cases were reported. Weekly reports declined by 75% (average of 11,367 weekly cases) in the 3 weeks from March 22, 2020, despite a tough nationwide lockdown being imposed in the weeks prior [21]. This failure was caused by a total number of variables, including a delay in data entry into the real-time national online TB monitoring E-system Nikshay, decreased use of health services, staff changes, and a drop in TB testing and findings. When associated with the number of cases discovered in February 2019, a similar 20% decrease was noted for February 2020. In comparison to the level of discovery before the pandemic, estimations show that the identification of TB cases worldwide reduced by an average of 25% during three months [26].

The year 2019 to 2021

The increase in COVID cases and resulting statewide lockdown were directly related to India's first fall in TB notifications in 2020. The Stop TB Partnership produced modeling research to study the possible impacts of the COVID-19 reaction on TB epidemiology. It predicts that every month of lockdown, there would be 2,32,665 more cases and 71,290 fatalities in India. This year, during the second wave of 2021, a comparable pattern is again noticeable, and a substantial decrease in the reporting of TB cases is observed. May 2021 saw India's lowest total number of TB cases in the previous three years [33,34].

A coin always has two sides to it. The number of TB cases would have decreased, which is a positive perspective. Due to masks, hand hygiene, and social seclusion, COVID-19 behavior was not only helpful in containing the pandemic but also resulted in a significant behavioral change in the Indian people, which may have contributed to limiting the spread of infection tuberculosis during the pandemic. The number of TB patients may be declining, and the second may have occurred because active TB notifications may not have been made owing to COVID-19 and the lockdown [33,35]. Based on Nikshay data, the effect of TB notification throughout the COVID-19 pandemic from 2019-2021 is shown in the 3 graphs below.

**Figure 3:**
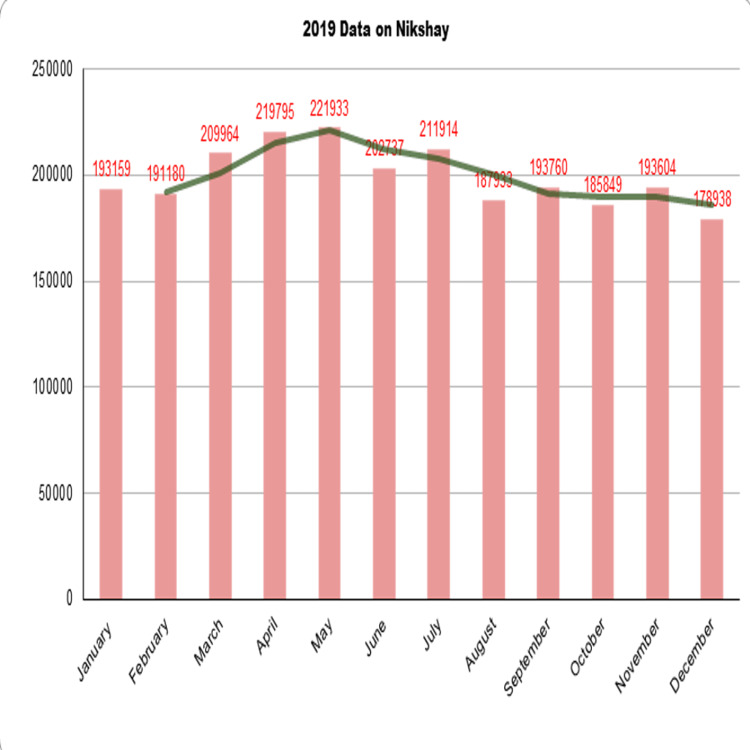
TB Notification in Nikshay Portal in 2019.

In the graph shown in Figure 3 below, the notification of Tb came down in December when COVID-19 infection entered India, which decreased even more in 2020.

**Figure 4:**
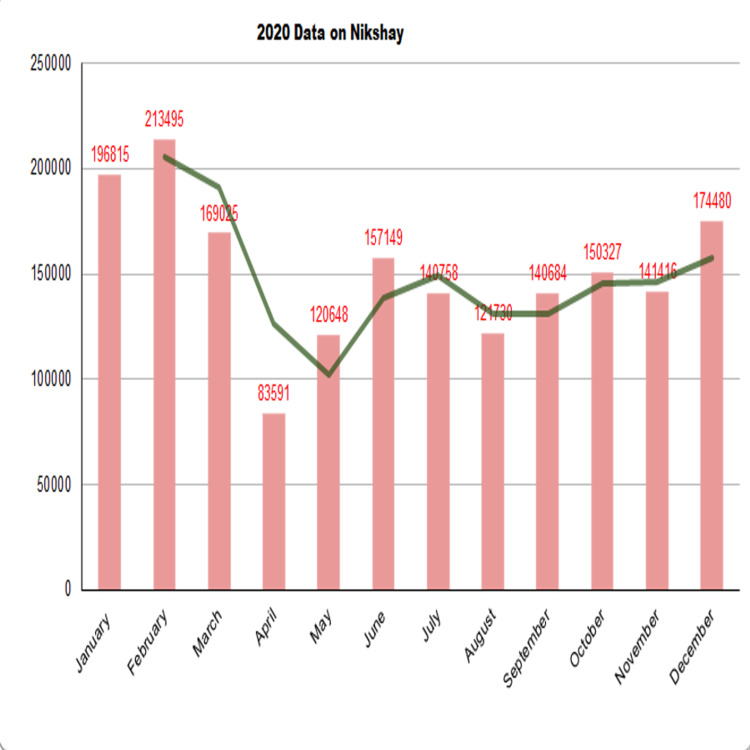
TB Notification in Nikshay Portal in 2020.

Before the COVID-19 infection spread over India in 2020, the TB notification rate was accurate for the year's first two months. However, as shown in Figure 4 below, the TB notification rate has declined since March and drastically reduced in April. There was a lockdown-like situation going on at that time. The impact of COVID-19 will be felt more in the TB notification area in 2020.

**Figure 5:**
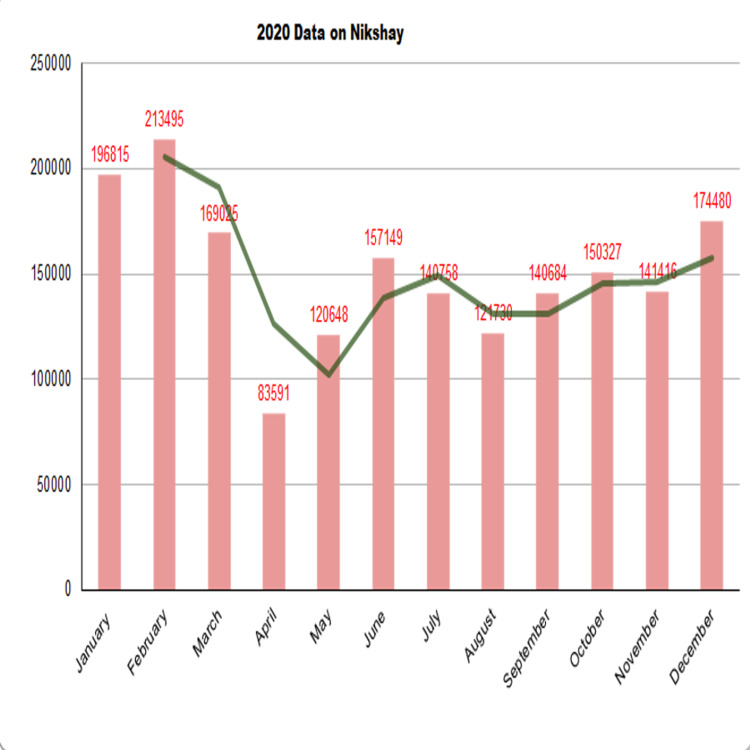
TB Notification in Nikshay Portal in 2021.

The notification of TB then decreased in 2021 as an effect of the second wave of COVID-19, as shown in Figure 5 below. The second COVID-19 spread over India and created a panic situation. A dramatic decrease in TB notifications occurred between April and March 2021.

The Health Ministry reported: "In 2020 and 2021, India paid Rs 670 crore for TB patients to complete the Direct Benefit Transfer system. In addition, the Honourable President of India launched the Pradhan Mantri TB Mukt Bharat Abhiyan in September 2022 to provide patients receiving TB treatment with additional nutritional support through community contributions from individuals and groups [36].

As an outcome of the country's TB programs reporting much fewer TB cases than previously, the danger of TB-related death has increased, amplifying the special effects of COVID-19 on the TB pandemic. Diagnoses for and availability of tuberculosis treatment are continuous problems with high-burden environments. During a historic and significant interruption like COVID-19, these problems will probably be compounded in their effects. Increases in TB-related mortality and long-term consequences on the burden of TB might arise from declines in TB findings, which would limit access to diagnosis and treatment. Over the following five years, a further 20% rise in the mortality rate from TB is predicted [24].

After March 24, 2020, As a result of the drastic actions taken in 2018 and 2019, the public and the promotion of TB case notification in the early part of 2020, when the national lockdown superseded all critical strategic interventions, India moved toward the goal of TB elimination. Significant progress has been achieved in the private sector [33,37].

## Conclusions

This review discusses how the reported TB case notifications in India from March 2020 to April 2021 differ from the hypothetical case notifications that would have been reported in the nonappearance of the pandemic period. There is a significant disparity between the number of TB cases reported during that time and the number estimated in the absence of a pandemic based on annual counts and cycle trends. As a result, there could be a protective impact on transmission in countries with a high TB burden where mask use is common. Furthermore, genetic epidemiology research has shown that up to 80% of TB spread occurs outside the house and that decreased mobility and increasing social distance may disrupt these transmission dynamics.

Both COVID-19 and TB are worldwide pandemics, with TB being a complicated problem to treat with present control methods since it affects not just India but the entire world. In some circumstances, like the evolution of resistance, the epidemic's various components are global, while in others, like the availability of infrastructure and transmission pathways, they are region-specific. As a result, a deeper comprehension of the dynamics of the disease at the community level is necessary for efficient global disease management.
